# Supplementary material for: Predicting visual function by interpreting a neuronal wiring diagram
Source: Nature. 2024 Oct 2;634(8032):113–23. doi: 10.1038/s41586-024-07953-5 (PMC11446822; doi:10.1038/s41586-024-07953-5)
Supplement: Supplementary file 6 — CRF and ERF predictions for individual TmY4 and TmY9 cells. Analogous to Supplementary Data 3, but for TmY target types. Shown are the top four monosynaptic pathways, the strongest pathway passing through each of the top ten intermediary types (ranking from Extended Data Fig. 7), and the trisynaptic pathway Tm1–TmY–Dm3–TmY (see the section entitled Prediction of spatial normalization). [file 41586_2024_7953_MOESM6_ESM.zip › DataS4/TmY9qΓèÑ/720575940620680640.pdf]

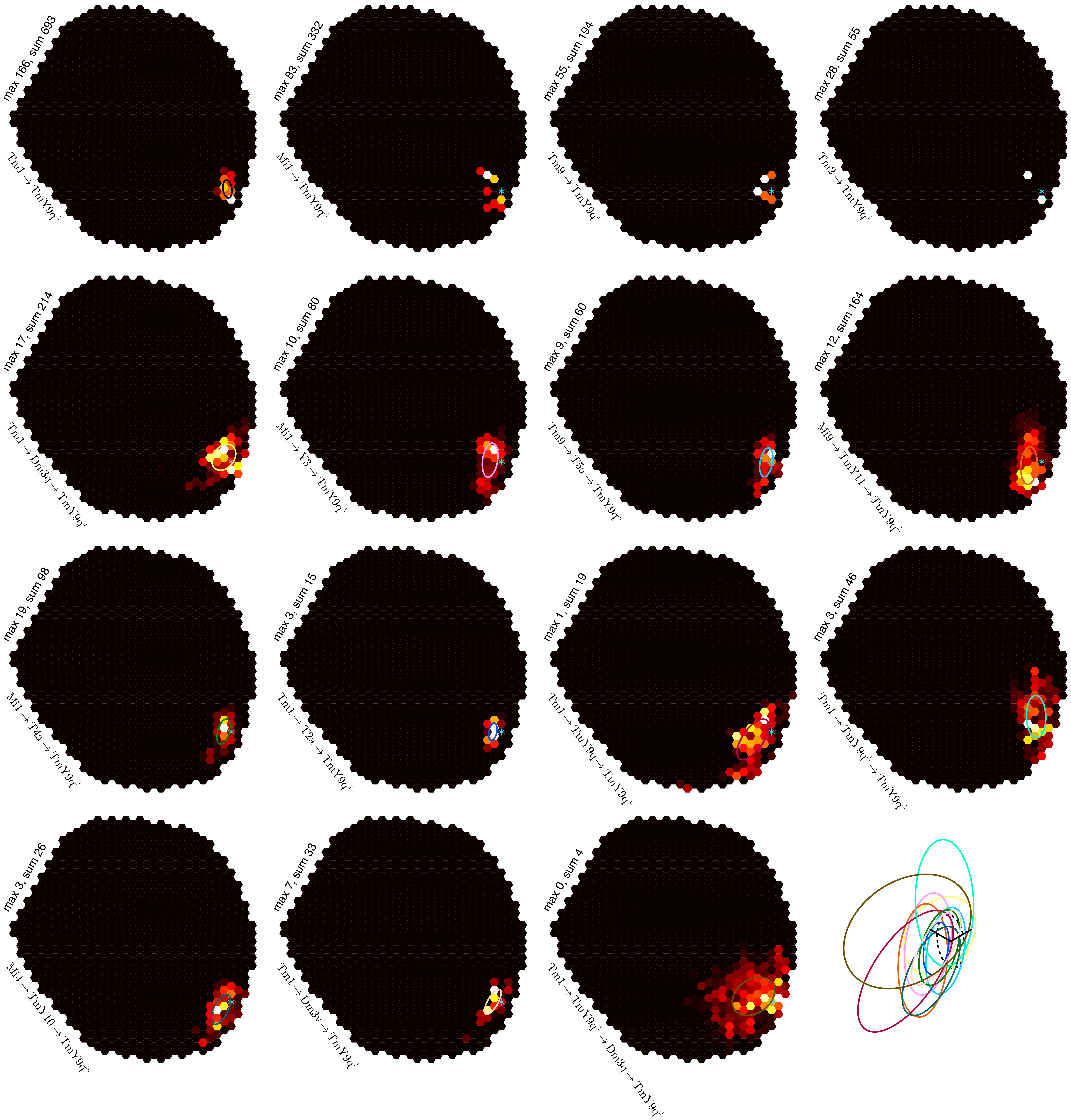

max 166, sum 693
Tm1 → TmY9q⊥
max 83, sum 332
Mi1 → TmY9q⊥
max 55, sum 194
Tm9 → TmY9q⊥
max 28, sum 55
Tm2 → TmY9q⊥
max 17, sum 214
Tm1 → Dm3q → TmY9q⊥
max 10, sum 80
Mi1 → Y3 → TmY9q⊥
max 9, sum 60
Tm9 → T5a → TmY9q⊥
max 12, sum 164
Mi9 → TmY11 → TmY9q⊥
max 19, sum 98
Mi1 → T4a → TmY9q⊥
max 3, sum 15
Tm1 → T2a → TmY9q⊥
max 1, sum 19
Tm1 → TmY9q → TmY9q⊥
max 3, sum 46
Tm1 → TmY9q⊥ → TmY9q⊥
max 3, sum 26
Mi4 → TmY10 → TmY9q⊥
max 7, sum 33
Tm1 → Dm3v → TmY9q⊥
max 0, sum 4
Tm1 → TmY9q⊥ → Dm3q → TmY9q⊥
